# Effects of Genetic Polymorphism in the *IFI27* Gene on Milk Fat Traits and Relevance to Lipid Metabolism in Bovine Mammary Epithelial Cells

**DOI:** 10.3390/ani14223284

**Published:** 2024-11-14

**Authors:** Xinyi Jiang, Zhihui Zhao, Xuanxu Chen, Fengshuai Miao, Jing Li, Haibin Yu, Ping Jiang, Ziwei Lin

**Affiliations:** 1 The Key Laboratory of Animal Genetic Resource and Breeding Innovation, College of Coastal Agricultural Sciences, Guangdong Ocean University, Zhanjiang 524088, China; jxy991130@163.com (X.J.); zhzhao@gdou.edu.cn (Z.Z.); chenxuanxugdou@163.com (X.C.); miaofengshuai@163.com (F.M.); lijing0305@outlook.com (J.L.); yuhb@gdou.edu.cn (H.Y.); jiangp@gdou.edu.cn (P.J.); 2 The Key Laboratory of Animal Resources and Breed Innovation in Western Guangdong Province, Zhanjiang 524088, China

**Keywords:** *IFI27*, SNP, milk fat, lipid metabolism, Chinese Holstein cow

## Abstract

Milk fat is an important indicator for evaluating milk quality and a symbol of the core competitiveness of the dairy industry. It can be improved through genetic and feed management factors. Interferon alpha-inducible protein 27 (*IFI27*) was found to be differentially expressed when comparing the transcriptome in high- and low-fat bovine mammary epithelial cells (bMECs) in our previous research. Therefore, this study aimed to investigate whether the IFI27 gene had a regulatory effect on lipid metabolism.We detected six SNPs in the *IFI27* gene (UTR-(-127) C>A, UTR-(-105) T>A, UTR-(-87) G>A, I1-763 G>T, E2-77 G>A, E2-127 G>T) in a Chinese Holstein cow population. Association analysis of the polymorphism of *IFI27* and milk quality traits showed that the AG and GG genotype of E2-77 G>A, and the GG and TT genotypes of E2-127 G>T were connected to milk fat (*p* < 0.05). Haplotype frequency analysis showed that H5H5 was associated with lower milk fat content (*p* < 0.05), while milk from H5H6 animals had a higher fat content (*p* < 0.05). Subsequently, *IFI27* overexpression vectors (PBI-CMV3-*IFI27*) and interference vectors (Pb7sk-GFP-shRNA) were constructed. Overexpression of the *IFI27* gene in bMECs caused a significant increase in triglycerides (TGs) content (*p* < 0.05) and decreases in cholesterol (CHOL) and nonestesterified fatty acid (NEFA) content (*p* < 0.05), while interference with *IFI27* expression produced opposing changes (*p* < 0.05). In summary, *IFI27* E2-77 G>A and *IFI27* E2-127 G>T may be useful as molecular markers in dairy cattle to measure milk fat, and the *IFI27* gene may play an important role in milk lipid metabolism.

## Simple Summary

In this study, six SNPs (UTR-(-127) C>A, UTR-(-105) T>A, UTR-(-87) G>A, I1-763 G>T, E2-77 G>A, E2-127 G>T) were detected in the IFI27 gene in a Chinese Holstein cow population. In addition, association analysis of the polymorphism of IFI27 and milk quality traits showed that the AG and GG genotypes of E2-77 G>A, and the GG and the TT genotypes of E2-127 G>T were connected to milk fat. Haplotype frequency analysis showed that individuals with an H5H6 genotype produced higher milk fat content than these with an H5H5 haplotype combination. Moreover, overexpression of the IFI27 gene in bovine mammary epithelial cells caused a significant increase in triglycerides content and a decrease in cholesterol and nonestesterified fatty acid content, while interference with IFI27 expression produced opposing changes. These results provide reference for the selection of milk fat traits in dairy cattle breeding and lay the foundation for further research of IFI27 gene function in milk fat metabolism.

## 1. Introduction

Milk and its products have become daily necessities to improve human health due to their high nutritional value and mellow taste. Milk quality traits mainly include milk fat and protein percentage, lactose, milk production, somatic cell count (SCC), and urea nitrogen (BUN). Among them, milk fat is easy to digest and absorb, and its composition and content are the main factors affecting the taste and nutritional value. Therefore, milk fat is one of the most important traits in dairy cow breeding. However, traditional breeding is too slow to meet the growing needs of consumers. 

Molecular markers, a reliable and effective scientific research technology, have been widely applied in animal genetic breeding and improved the milk production performance of dairy cows [1,2,3]. Single nucleotide polymorphisms (SNPs) have the advantages of good genetic stability, high accuracy, and easy automation of analysis. Currently, SNPs are widely used as molecular marker techniques in cow breeding, selecting candidate genes that are significantly related to economic factors such as milk fat, providing evidence for further research on the function of these genes [4]. A study reported that six SNPs of the long-chain acyl-CoA synthetase 1 (*ACSL1*) gene in Chinese Holstein cows were associated with milk yield, milk fat and protein content, and somatic cell score (SCS) [5]. Li et al. [6] revealed that the SNP c.908 C>T had significant effects that included increasing milk fat and protein yield, indicating the key role of the fatty acid desaturase 2 (*FADS2*) gene in influencing milk production traits. Du et al. [4] found that a total of 21 SNPs identified in the pyruvate kinase L/R (*PKLR*) gene were related to milk yield and milk fat. Therefore, SNPs can serve as candidate genetic markers for molecular breeding of dairy cattle to select target traits [7,8,9].

Interferon alpha-inducible protein 27 (*IFI27*), also known as the interferon-stimulated gene 12a (*ISG12a*), is located on chromosome 14q32 [10]. The *IFI27* gene is involved in various biological processes, including pathogenesis of various viral infections, apoptosis, and innate immunity [11,12,13,14]. Our previous transcriptome analysis found that the *IFI27* was gene differentially expressed in high- and low-fat bovine mammary epithelial cell lines (BMECs) from Chinese Holstein cows [15]. However, the effects of *IFI27* on lipid metabolism had not then been clarified. Therefore, this current study aimed to detect the SNPs of the *IFI27* in Chinese Holstein cows to investigate the association with genotypes and milk fat traits. In addition, in this study, the vector construction of IFI27 was used to reveal the potential effects of *IFI27* in bovine mammary epithelial cells (bMECs) on milk lipid metabolism. The results of this research identify potential markers of milk fat traits to support future marker-assisted selection in dairy cow breeding and production. Meanwhile, this study lays the foundation for further study of the *IFI27* function in milk lipid metabolism.

## 2. Materials and Methods

### 2.1. Animals and Milk Traits Analysis

In this study, 132 Chinese Holstein cows with similar genetic backgrounds were provided by a dairy farm in Heilongjiang Province. Milk was from the cow’ second pregnancies and collected every 30 days for a total of 11 times. Following sample collection, milk quality was tested and data were collected by a milk quality analyzer (FOSS, MilkoScan FT3, Zealand, Denmark).

### 2.2. Animal Cell Line

The mammary epithelial cells of Chinese Holstein cows were provided by the Laboratory of Molecular Genetics at Guangdong Ocean University. The experimental procedures were conducted in accordance with the Guide for Guangdong Ocean University Ethics Committee.

### 2.3. Primers Design and Polymerase Chain Reaction (PCR) Amplification

According to the existing published sequence of the bovine *IFI27* gene (ENSBTAT000004093.5), SNP primers and *IFI27* gene CDS primers were designed using Premier 5 software. The short hairpin RNA (shRNA) primers were designed using the BLOCK-iT^™^ RNAi Designer: http://rnaidesigner.thermofisher.com/rnaiexpress/insert.do (accessed on 10 May 2022). The primers were synthesized by BBI (Guangzhou, China). The primers’ sequences are provided in Table 1.

For PCR amplification, a total of 20 µL of 10 pmol·L^−1^ of each primer was combined with 140 ng DNA, 5 µL dNTP mix, 2 µL buffer, and 1.5 µL Taq DNA polymerase, and then distilled H_2_O was added to 20 µL. Firstly, the PCR mixture was incubated at 95 °C for 5 min and 35 cycles of 95 °C for 30 s. Secondly, each fragment was annealed for 30 s, 1000 bp min^−1^ at 62 °C, and finally extended at 72 °C for 10 min.

**Figure 1:**
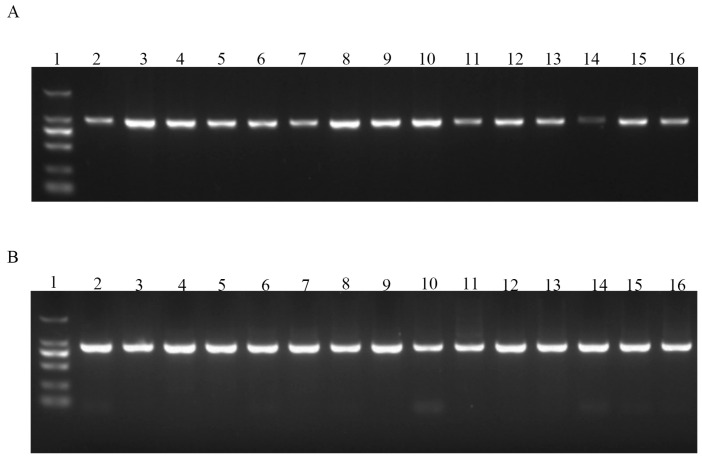
Gel electrophoresis pictures: (**A**) gel electrophoresis of the first pair of polymorphism primers: lane 1 is the DNA marker; lane 2 to lane 6, UTR-(-127); lane 7 to 11, UTR-(-105); lane 12 to 16, UTR-(-87); (**B**) gel electrophoresis of the second pair of polymorphism primers: lane 1 is the DNA marker; lane 2 to lane 6, I1-763; lane 7 to 11, E2-77; and lane 12 to 16, E2-127.

The DNA size of PCR products was detected by a gel electrophoresis system. In brief, 5 µL DNA was run on a 1.5% agarose gel at 100 V for 30 min. The gel was UV visualized with a Tanon 1220 Gel Image System (Figure 1). 

### 2.4. DNA Extraction

DNA extraction was performed using a TIANamp Blood DNA Kit (TIANGEN, Beijing, China). A Nanodrop Lite spectrophotometer (Thermo Scientific, Waltham, MA, USA) was used to determine the concentration of the DNA. DNA quality was measured by agarose gel electrophoresis.

### 2.5. SNPs Detection of the IFI27 Gene

PCR amplification was carried out on the DNA samples of 132 Chinese Holstein cows. The primer’ sequences and the reaction system of SNP are given in the method in Section 2.3. The PCR products were sent to BBI (Guangzhou, China) for sequencing. The polymorphisms of the key functional region of the *IFI27* gene were determined through Sanger sequencing.

### 2.6. Correlation Analysis

DNASTAR SeqMan software 7.1.0 (44.1) was used to observe the overlapping peaks in the sequencing results. The formula was used to calculate the frequency of genotypes and the gene frequency of alleles. The genetic heterozygosity (He) and the numbers of effective alleles (Ne) were calculated with Popgene32 software. PIC0.6 software was used to calculate polymorphic information content (PIC). The X^2^ value was calculated by the formula for Hardy–Weinberg balance detection. Haploview 4.2 software was used to analyze the linkage–unbalance relationships of SNP sites. Multiple comparison of single-factor variance was used in SPSS 23.0 software was used to analyze the correlations between different genotypes and haplotypes of SNP sites and milk fat traits.

### 2.7. Construction of pBI-CMV3-IFI27 and pb7sk-GFP-shRNA

The CDS of *IFI27* with *Hind*III and *Mlu*I restriction sites were obtained using PCR. The pBI-CMV3 vector was linearized with *Hind*III and *Mlu*I (New England Biolabs, Ipswich, MA, USA). Then, the CDS of the *IFI27* gene was cloned into a linearized pBI-CMV3 plasmid using T4 ligase (Thermo Scientific, Waltham, MA, USA).

The shRNA primers of *IFI27* were annealed to form double-stranded RNA. The pPb7sk-GFP-Neo vector was linearized with *Bbs*I and *Bam*HI (New England Biolabs, Ipswich, MA, USA), and double-stranded RNA was then cloned into the linearized pPb7sk-GFP-Neo plasmid by T4 ligase (Thermo Scientific).

### 2.8. bMECs Culture and Treatment

The bMECs were approximately 80–90% confluent on the day of transfection in six-well plates. On the day of transfection, diluted 3.5 µg DNA and 6 µL of viafect transfection reagent (Promega, Madison, WI, USA) were mixed lightly with 200 µL Opti-MEM serum-free media (Sigma-Aldrich, St. Louis, MO, USA). The mixture was incubated at room temperature for 20 min and added into the well plate. The green fluorescent protein (GFP) expression was measured with a fluorescence microscope (NikonTE2000, Tokyo, Japan).

### 2.9. RT-qPCR

The RNA extraction of bMECs used Trizol reagent (Thermo Fisher Scientific, MA, USA). Then chloroform, isopropanol, and 75% ethanol were sequentially added to extract and purify the RNA. The cDNA synthesis was carried out using a Prime-Script^™^ RT reagent kit (TaKaRa, Bejing, China). Then, expression of *IFI27* genes was quantitated using a SYBR Green RT-qPCR MasterMix (TaKaRa). The primers used for the PCR are listed in Table 2. The 2^−∆∆CT^ method was used for comparative quantification.

### 2.10. ELISA

The extraction of total protein was performed using radio immunoprecipitation assay (RIPA) lysis buffer (TaKaRa). The cells were incubated on ice for 20 min and then centrifuged at 4 °C with 12,000 r for 30 min to remove the lysate. The protein level was determined using a BovineIFI27 ELISA Kit (Meimian Industrai Co. Ltd., Jiangsu, China).

### 2.11. Determination of TGs, CHOL, and NEFA Content in bMECs of IFI27 Gene

A triglyceride detection kit and cholesterol assay kit (Applygen Technologies, Beijing, China) and a nonestesterified fatty acid detection kit (NanJing JianCheng Bioengineering Institute, Nanjin, China) were utilized to measure the TGs, CHOL, and NEFA content. The cellular contents of TG, CHOL, and NEFA were normalized by protein content. An Enhanced BCA Protein Quantitation Assay Kit (KeyGEN BioTECH, Jiangsu, China) was used to detect the total protein concentrations.

### 2.12. Statistical Analysis

GraphPad Prism 10.1.2 (GraphPad Software Inc., San Diego, CA, USA) was used to analyze data. A completely randomized Student’s *t*-test in SPSS software (IBM Corporation, Armonk, NY, USA) was used for group comparisons. All data were presented as mean ± standard error of mean (SEM). The statistical significance was expressed as (*) *p* < 0.05 and (**) *p* < 0.01.

## 3. Results

### 3.1. Six Polymorphisms Were Found in IFI27 Genes

**Figure 2:**
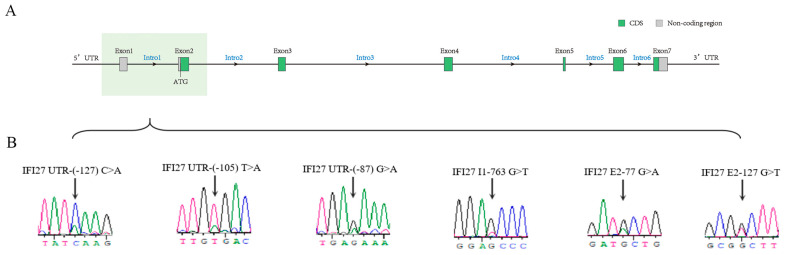
Analysis and sequencing of SNPs in the *IFI27* gene: (**A**) identification of SNPs in the key functional domains of the *IFI27* gene; (**B**) six SNP sites of *IF27*.

As shown in Figure 2, at locations 127 bp,105 bp, and 87 bp in the 5′UTR, there were C>A, T>A, and G>A substitutions (*IFI27* UTR-(-127) C>A, *IFI27* UTR-(-105) T>A, and *IFI27* UTR-(-87) G>A). Additionally, there was a substitution of G>T (*IFI27* I1-763 G>T) at location 763 bp in the first intron. Furthermore, there were G>A and G>T substitutions (*IFI27* E2-77 G>A and *IFI27* E2-127 G>T) at locations 77 bp and 127 bp in the second exon. Accordingly, six polymorphisms were found in the UTR, intron, and exon regions of the bovine *IFI27* gene.

### 3.2. Genetic Diversity of SNPs in the IFI27 Gene

Table 3 shows the SNPs’ genetic diversity in the Chinese Holstein cows. The PIC values of UTR-(-127) C>A, E2-77-G>A, and E2-127 G>T were between 0.25 and 0.5, while the PIC values of UTR-(-105) T>A, UTR-(-87) G>A and I1-763 G>T were less than 0.25. This indicated UTR-(-127) C>A, E2-77 G>A, and E2-127 G>T belonged to the category of medium polymorphic loci, while UTR-(-105) T>A, UTR-(-87) G>A and I1-763 G>T were low polymorphic loci. The results of X^2^ testing showed that except for the E2-127 G>T, whose X^2^ was greater than 9.21 (*p* < 0.01), the X^2^ values for the rest of the loci were less than 5.99 (*p* > 0.05). This indicated that the gene and genotype frequencies of UTR-(-127) C>A, UTR-(-105) T>A, UTR-(-87) G>A, I1-763 G>T, and E2-77 G>A did not differ significantly among the populations and were in Hardy–Weinberg equilibrium.

### 3.3. The Association of IFI27 Polymorphisms with Milk Quality

The association between *IFI27* gene polymorphisms and milk quality traits is presented in Table 4. We focused on the association of *IFI27* polymorphisms with milk fat rate.

As shown in Table 4, the E2-77 G>A and E2-127 G>T of the *IFI27* gene were potentially associated with milk fat rate (*p* < 0.05). AG genotype substitution of the E2-77 G>A led to a higher milk fat rate and the GG genotype had a lower milk fat rate (*p* < 0.05). Compared with the GT genotype of E2-127 G>T SNP, the GG genotype had a higher milk fat rate and TT genotype had a lower milk fat rate (*p* < 0.05).

### 3.4. The Linkage Analysis of IFI27 Polymorphisms Haplotype and Milk Quality

**Figure 3:**
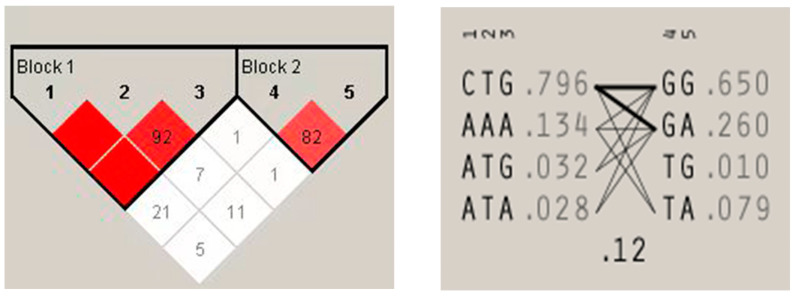
Linkage and haplotype analyses of SNPs of *IFI27* gene. Block1 with red color presents strong linkage between UTR-(-127) C>A(1), UTR-(-105) T>A(2), and UTR-(-87) G>A(3); four haplotypes are shown, with haplotype frequency. Block2 with red color presents strong linkage between I1-763 G>T(4) and E2-77 G>A(5). A total of 9 haplotype combinations with biological repetition significance (number of individuals ≥ 3) were composed.

The linkage analysis of six SNPs in *IFI27* is shown in Figure 3. Strong linkage relationships were observed between UTR-(-127) C>A, UTR-(-105) T>A, and UTR-(-87) G>A, as well as between I1-763 G>T and E2-77 G>A. A total of nine haplotype combinations with biological repetition significance (number of individuals ≥ 3) were composed. 

The association between *IFI27* gene polymorphisms haplotype and milk quality traits is shown in Table 5. Individuals with an H5H6 (4.60 ± 0.04) genotype had milk with a higher fat content than these with an H5H5 (4.42 ± 0.04) haplotype combination (*p* < 0.05).

### 3.5. Construction and Transfection of pBI-CMV3-IFI27 and pb7sk-GFP-shRNA Vector

**Figure 4:**
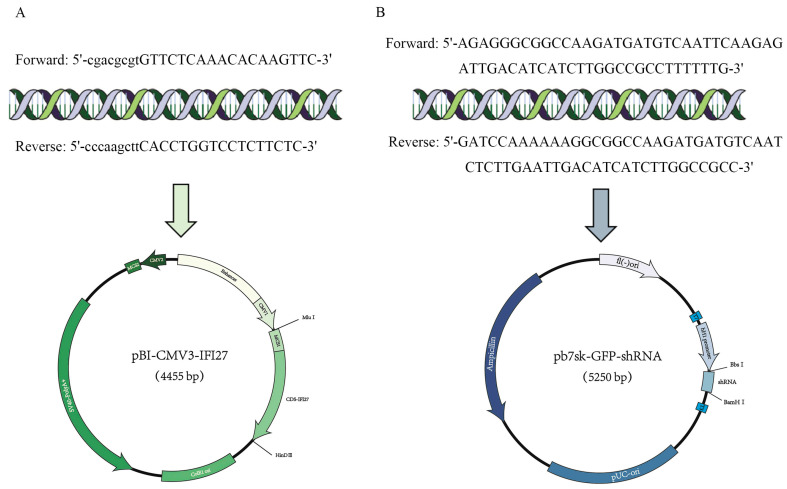
*IFI27* gene interference vectors and overexpression vectors: (**A**) primer sequence and overexpression vectors (pBI-CMV3-*IFI27*); (**B**) primer sequence of the RNA interference target sequence and interference vectors (pb7sk-GFP-shRNA4).

DNA fragments obtained through PCR and pBI-CMV3 plasmid, including the *IFI 27* coding sequence (CDS), were selected as vectors for overexpression of *IFI27* (Figure 4A). Moreover, shRNA targeting the oligonucleotide sequence of the *IFI27* gene was cloned and constructed into *Bbs*I and *Bam*HI sites of the pb7sk-GFP-Neo vector (Figure 4B).

**Figure 5:**
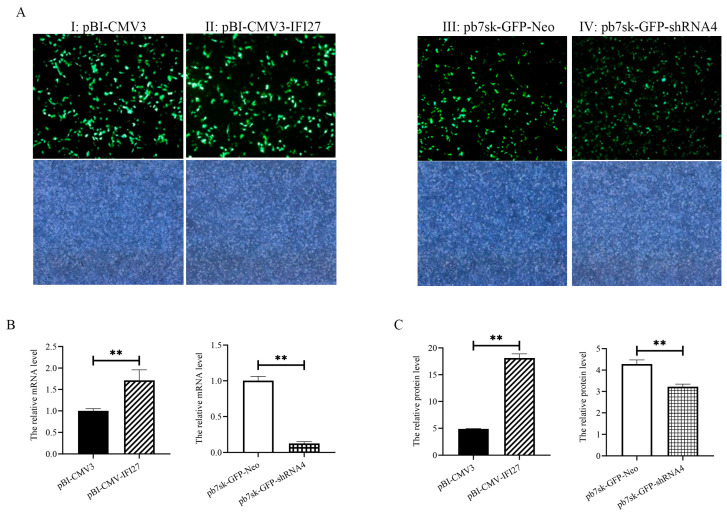
Expression of *IFI27* in two vector groups: (**A**) green fluorescence protein expression observation by fluorescent microscope. pBI-CMV3 refers to bMECs transfected with pBI-CMV3 vector; pBI-CMV3-IFI27, bMECs transfected with pBI-CMV3-*IFI27* vector; pb7sk-GFP-Neo, bMECs transfected with pb7sk-GFP-Neo vector; pb7sk-GFP-shRNA4, bMECs transfected with pBI-CMV3-IFI27vector; (**B**) mRNA expression of *IFI27* in bMECs; (**C**) protein expression of IFI27 in bMECs. ** p < 0.01.

As illustrated in Figure 5A, green fluorescence protein expression indicated the successful transfection of plasmid into bMECs. Compared with the pBI-CMV3 group, the mRNA and protein expression of IFI27 in the pBI-CMV3-*IFI27* group were markedly increased (*p* < 0.01) (Figure 5B,C). Moreover, in the pb7sk-GFP-shRNA4 group, there was a trend of lower mRNA and protein expression of IFI27 compared with the control group (*p* < 0.01, Figure 5B,C).

### 3.6. The IFI27 Gene Increases the Triglycerides (TGs) Content and Decreases the Cholesterol (CHOL) and Nonestesterified Fatty Acid (NEFA) Content in bMECs

**Figure 6:**
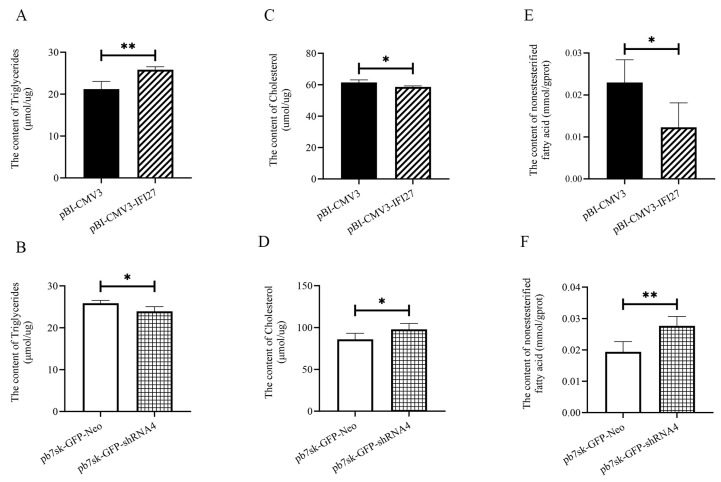
The TGs, CHO, and NEFA contents of each transfected group in bMECs: (**A**,**C**,**E**) The TGs, CHOL, and NEFA contents in the pBI-CMV3-*IFI27* group; (**B**,**D**,**F**) The TGs, CHO, and NEFA contents in the pb7sk-GFP-shRNA4 group. pBI-CMV3 refers to bMECs transfected with pBI-CMV3 vector; pBI-CMV3-IFI27, bMECs transfected with pBI-CMV3-IFI27 vector; pb7sk-GFP-Neo, bMECs transfected with pb7sk-GFP-Neo vector; pb7sk-GFP-shRNA4, bMECs transfected with pBI-CMV3-IFI27vector. Error bars indicate SEM.* *p* < 0.05, ** *p* < 0.01.

The TGs, CHOL, and NEFA content of bMECs were evaluated after the transfection of pBI-CMV3-*IFI27* and pb7sk-GFP-shRNA4 (Figure 6). TGs content increased when *IFI27* gene overexpressed (*p* < 0.05, Figure 5A), while CHOL and NEFA decreased (*p* < 0.05, Figure 6C,E).

Compared with the pb7sk-GFP-Neo group, the TGs content of the pb7sk-GFP-shRNA4 group markedly decreased (*p* < 0.05, Figure 6B), and CHOL and NEFA showed an upward trend in the pb7sk-GFP-shRNA4 group (Figure 6D,F).

## 4. Discussion

Chinese Holstein are an important breed of dairy cattle in China and are characterized by their tall size, high milk production, and gentle temperament. The milk quality traits of Chinese Holstein cows (milk fat percentage, milk protein percentage, lactose, milk production, etc.) are important economic traits that are regulated by many functional genes and complex genetic mechanisms. Thus, the breeding of excellent Holstein cows is of great research value. Milk fat is a good source of dietary fat. Recently, consumer demand and preference for milk fat have changed, thereby encouraging researchers to explore mechanisms of producing dairy products with different fat contents [15,16]. In this study, Sanger sequencing technology was used to locate polymorphisms of *IFI27* in Chinese Holstein cow to determine whether there were molecular markers that could be used to detect their milk quality traits. Furthermore, in our previous study, transcriptome analysis indicated differential expression of *IFI27* in the mRNA expression between high- and low-fat BMECs [15]. Therefore, we focused particularly on the regulatory effect of the *IFI27* gene on bovine lipid metabolism.

In this study, six SNPs (UTR-(-127) C>A, UTR-(-105) T>A, UTR-(-87) G>A, I1-763 G>T, E2-77 G>A, and E2-127 G>T) in the *IFI27* gene were completely identified. Among the six SNPs, UTR-(-127) C>A, E2-77-G>A, and E2-127 G>T were moderate polymorphisms in terms of population that could be well utilized for selection. In addition, UTR-(-127) C>A, UTR-(-105) T>A, UTR-(-87) G>A, I1-763 G>T, and E2-77 G>A were in Hardy–Weinberg equilibrium, indicating that these individuals have been less affected by artificial selection and the population has better heritability. In addition, the milk fat content from the individuals with the AG genotype at the E2-77 G>A and the GG genotype at the E2-127 G> T was dramatically higher than that of other SNPs, which may suggest that the AG genotype of the E2-77 G>A locus and the GG genotype of the E2-127 G>T locus can be used as the optimal genotypes for improving milk fat content in dairy cows. Compared with SNP analysis, haplotype analysis can provide a more accurate statistical effect in association research regarding complex traits. This research found that the milk fat content of the H5H6 haplotype was the highest, and the milk fat content of H5H5 was the lowest. Different consumers have different needs in terms of content and proportion of milk fat. For example, children need high milk fat for growth, while cardiovascular and cerebrovascular patients and diabetes patients need low-fat or skimmed milk. Therefore, the H5H6 and H5H5 haplotype combination may be used as a haplotype combination to regulate the milk fat content in dairy cow milk. Our study indicated that these two SNPs and the combined haplotypes might be molecular markers for the detection of milk fat content in dairy cow milk. Therefore, we focused on the relationship between *IFI27* and lipid metabolism. 

To further analyze the *IFI27* gene’s function on milk lipid metabolism, the overexpression and interference vector of the *IFI27* gene were constructed and transfected into bMECs. Milk fat is mainly composed of triglycerides (98%) in addition to two types of acylglycerols, cholesterol, phospholipids, and free fatty acids [17]. In this study, it was found that overexpression of the *IFI27* gene in bMECs resulted in a significant increase in intracellular TGs content and a decrease in CHOL and NEFA content, while interference with *IFI27* expression caused opposing changes. TGs, an indicator of dairy product quality, play an important role in energy storage. Interestingly, a study demonstrated that lipopolysaccharide (LPS) markedly reduced the TGs and NEFA content in bMECs, compared with the control group [18]. Hence, we speculated that the *IFI27* gene might have an effect on intracellular NEFA and TGs content when Chinese Holstein cows suffer from mastitis. CHOL exists in almost all cells of the human body and is involved in maintaining the functions of organisms [19]. However, CHOL has a bidirectional (both good and bad) effect in the body [20]. The World Health Organization and the American Heart Association have suggested that people should decrease their intake of saturated fatty acids and CHOL to reduce the risk of coronary heart disease. Therefore, there is a demand in cow breeding for low-cholesterol dairy products. In our study, overexpression of the *IFI27* gene in bMECs resulted in a decrease in CHOL, while interference with *IFI27* expression led to an increase in CHOL. These results indicate that the *IFI27* gene may be a target gene to meet the demands of consumers in terms of different milk fat content.

Therefore, further study of the fat composition of cow milk is not only beneficial in the genetic screening of dairy cattle, but can also help us to meet the needs of different consumers. This is of great economic significance for the development of the market for milk and dairy products.

## 5. Conclusions

To sum up, there are six SNPs of the *IFI27* gene, namely, UTR-(-127) C>A, UTR-(-105) T>A, UTR-(-87) G>A, I1-763 G>T, E2-77 G>A, E2-127 G>T. Among these, E2-77 G>A and E2-127 G>T are related to milk fat traits in Chinese Holstein cows. These two SNPs may serve as effective molecular markers that could be utilized for marker-assisted selection related to milk fat traits. Furthermore, pBI-CMV3-*IFI27* and pb7sk-GFP-shRNA vectors were constructed. The *IFI27* expression in bMECs after interference and overexpression can regulate TGs, CHOL, and NEFA content, which are relevant to lipid metabolism. Therefore, this research lays the foundation for studying the mechanism of the *IFI27* gene in the lipid metabolism in dairy cows.

## Figures and Tables

**Table 1:** Primer sequences used in the experiment.

Primer		Forward Sequences	Reverse Sequences	Target Sequence	Amplified Fragment (bp)	Annealing Temperature (°C)
shRNA of *IFI27*		5′-AGAGGGCGGCCAAGATGATGTCAATTCAAGAGATTGACATCATCTTGGCCGCCTTTTTTG-3′	5′-GATCCAAAAAAGGCGGCCAAGATGATGTCAATCTCTTGAATTGACATCATCTTGGCCGCC-3′	GGCGGCCAAGATGATGTCAAT	---	---
Polymorphism	UTR-(-127) C>A, UTR-(-105) T>A, UTR-(-87) G>A	5′-AGCAGAGAAAGGTATGTGGCAG-3′	5′-AGTACACGGGAACTGATACAGG-3′	---	958	60
	I1-763 G>T, E2-77 G>A, E2-127 G>T	5′-CTTCCCAAGCCCGCAT-3′	5′-GGAAATGGACCTGAATTGAAG-3′	---	896	60
Coding region of *IFI27*		5′-cgacgcgtGTTCTCAAACACAAGTTC-3′	5′-cccaagcttCACCTGGTCCTCTTCTC-3′	---	657	60

**Table 2:** Primer sequences used for RT-qPCR.

Primer	Forward Sequences	Reverse Sequences	Sequence Number	Amplified Fragment (bp)
*IFI27*	5’-TGAGCACTTTGCCAGTAGGAG-3’	5’-CCAAGGAGGAGGCAGTGAT-3’	NM_001038050.2	657
β-actin	5’-AGAGCAAGAGAGGCATCC-3’	5’-TCGTTGTAGAAGGTGTGGT-3’	NM_173979.3	133

**Table 3:** Genetic diversity of the bovine *IFI27* gene.

Type	Frequency					He	Ne	PIC	X^2^
	Genotype Frequency			Allele Frequency					
UTR-(-127) C>A	CC(0.60)	CA(0.40)	---	C(0.80)	A(0.20)	0.32	1.47	0.27	2.45
UTR-(-105) T>A	TT(0.72)	TA(0.28)	---	T(0.86)	A(0.14)	0.24	1.32	0.21	0.74
UTR-(-87) G>A	GG(0.67)	GA(0.33)	---	G(0.84)	A(0.16)	0.27	1.38	0.34	1.52
I1-763 G>T	GG(0.82)	GT(0.18)	---	G(0.91)	T(0.09)	0.17	1.20	0.15	0.23
E2-77 G>A	AA(0.13)	AG(0.41)	GG(0.46)	A(0.33)	G(0.67)	0.44	1.80	0.35	0.64
E2-127 G>T	GG(0.43)	GT(0.18)	TT(0.39)	G(0.52)	T(0.48)	0.50	2.00	0.37	50.70

He refers to desired heterozygosity, Ne refers to effective allele number, and PIC refers to polymorphism information content. X^2^ represents the difference between gene frequency and genotype frequency, X^2^_0.05_(df = 2) = 5.99, X^2^_0.01_(df = 2) = 9.21.

**Table 4:** The association of the six SNPs in the *IFI27* gene with milk quality traits.

SNP Genotype		Milk Yield (kg)	Fat (%)	Protein (%)	Lactose (%)	Dry Matter (%)	SCC (10^4^ mL^−1^)	BUN (mg/L)	FCM (kg)
UTR-(-127) C>A	CC	27.41 ± 0.66	4.50 ± 0.04	3.42 ± 0.02	4.79 ± 0.02	13.53 ± 0.08	34.91 ± 5.26	19.13 ^b^ ± 0.20	37.97 ± 1.12
	CA	26.89 ± 0.92	4.55 ± 0.05	3.50 ± 0.03	4.80 ± 0.02	13.69 ± 0.11	53.07 ± 10.57	19.81 ^a^ ± 0.22	41.16 ± 1.67
UTR-(-105) T>A	TT	27.39 ± 0.60	4.50 ± 0.04	3.44 ± 0.02	4.79 ± 0.02	13.55 ± 0.07	38.72 ± 5.63	19.19 ^b^ ± 0.17	38.46 ± 1.03
	TA	26.72 ± 1.14	4.58 ± 0.06	3.49 ± 0.04	4.79 ± 0.02	13.70 ± 0.13	51.17 ± 12.37	19.96 ^a^ ± 0.27	41.26 ± 2.13
UTR-(-87) G>A	GG	27.33 ± 0.60	4.50 ± 0.04	3.44 ± 0.02	4.79 ± 0.02	13.55 ± 0.07	34.04 ^b^ ± 4.75	19.21 ± 0.18	38.20 ± 1.05
	GA	26.94 ± 1.09	4.56 ± 0.06	3.48 ± 0.04	4.79 ± 0.03	13.67 ± 0.12	59.02 ^a^ ± 12.70	19.80 ± 0.25	41.41 ± 1.92
I1-763 G>T	GG	26.82 ± 0.52	4.50 ± 0.03	3.42 ^b^ ± 0.02	4.78 ^a^ ± 0.02	13.49 ± 0.07	41.62 ± 5.38	19.40 ± 0.15	38.83 ± 0.95
	GT	28.03 ± 1.26	4.60 ± 0.07	3.53 ^a^ ± 0.04	4.70 ^b^ ± 0.04	13.80 ± 0.14	44.55 ± 10.49	19.33 ± 0.30	39.20 ± 1.95
E2-77 G>A	AA	27.04 ± 1.69	4.54 ± 0.10	3.45 ± 0.05	4.75 ± 0.05	13.53 ± 0.20	51.76 ± 15.08	19.49 ± 0.36	37.68 ± 3.27
	AG	26.92 ± 0.76	4.60 ^a^ ± 0.04	3.48 ^a^ ± 0.03	4.76 ± 0.02	13.76 ± 0.08	38.66 ± 6.03	19.44 ± 0.21	39.39 ± 1.22
	GG	25.81 ± 0.99	4.22 ^b^ ± 0.13	3.23 ^b^ ± 0.10	4.54 ± 0.14	12.70 ± 0.38	40.54 ± 7.61	18.37 ± 0.57	36.89 ± 1.58
E2-127 G>T	GG	26.82 ± 0.79	4.58 ^a^ ± 0.05	3.47 ^a^ ± 0.02	4.75 ± 0.02	13.70 ^a^ ± 0.09	42.59 ± 6.36	19.28 ± 0.20	38.13 ± 1.37
	GT	26.61 ± 1.23	4.48 ± 0.06	3.46 ± 0.06	4.77 ± 0.03	13.52 ± 0.14	45.15 ± 11.92	19.67 ± 0.32	38.25 ± 2.03
	TT	25.90 ± 1.10	4.20 ^b^ ± 0.15	3.19 ^b^ ± 0.11	4.50 ± 0.16	12.62 ^b^ ± 0.45	37.95 ± 8.16	18.26 ± 0.66	37.74 ± 1.78

Different lowercase letters (a, b) in the same column show significant differences (*p* < 0.05) between the mean values of the traits. Absence of lowercase letters indicates no significant difference (*p* > 0.05) between the mean values of the traits. Test data are presented as “mean ± s standard error of mean (SEM)”.

**Table 5:** Relationships between haplotype combinations in the *IFI27* gene and milk quality traits.

Haplotype Combination	Milk Yield (kg)	Fat (%)	Protein (%)	Lactose (%)	Dry Matter (%)	SCC (10^4^ mL^−1^)	BUN (mg/L)	FCM (kg)
H1H1	27.41 ± 0.66	4.50 ± 0.04	3.42 ± 0.02	4.79 ± 0.02	13.53 ± 0.08	34.91 ^b^ ± 5.26	19.13 ^b^ ± 0.20	37.97 ± 1.12
H1H2	26.75 ± 1.18	4.58 ± 0.06	3.49 ± 0.04	4.79 ± 0.02	13.68 ± 0.13	52.62 ± 12.72	19.91 ^a^ ± 0.28	41.28 ± 2.20
H1H3	26.77 ± 1.59	4.47 ± 0.13	3.52 ± 0.10	4.83 ± 0.04	13.73 ± 0.29	29.57 ^b^ ± 8.22	19.67 ± 0.55	39.96 ± 3.83
H1H4	27.86 ± 2.99	4.45 ± 0.18	3.47 ± 0.12	4.76 ± 0.10	13.60 ± 0.38	89.97 ^a^ ± 42.28	19.23 ± 0.52	42.04 ± 3.93
H5H5	27.31 ± 0.66	4.42 ^b^ ± 0.04	3.38 ^b^ ± 0.03	4.79 ± 0.03	13.33 ± 0.10	40.35 ± 7.76	19.35 ± 0.20	38.84 ± 1.21
H5H6	26.17 ± 0.85	4.60 ^a^ ± 0.04	3.46 ± 0.03	4.77 ± 0.02	13.71 ± 0.09	37.59 ± 6.99	19.55 ± 0.25	38.97 ± 1.54
H5H7	28.77 ± 1.50	4.59 ± 0.08	3.52 ^a^ ± 0.06	4.72 ± 0.04	13.89 ± 0.18	41.31 ± 12.20	19.19 ± 0.39	40.41 ± 1.88
H6H6	26.47 ± 2.34	4.52 ± 0.15	3.46 ± 0.08	4.79 ± 0.04	13.58 ± 0.30	63.64 ± 23.17	19.19 ± 0.51	38.30 ± 4.38
H6H7	27.98 ± 2.48	4.58 ± 0.10	3.44 ± 0.04	4.69 ± 0.10	13.45 ± 0.25	31.96 ± 9.19	19.97 ± 0.41	36.64 ± 5.24

Different lowercase letters (a, b) in the same column show significant differences (*p* < 0.05) between the mean values of the traits. Absence of lowercase letters indicates no significant difference (*p* > 0.05) between the mean values of the traits. Test data are expressed as “mean ± SEM”.

## Data Availability

Data available on request due to restrictions.

## References

[1] Fang M., Fu W., Jiang D., Zhang Q., Sun D., Ding X., Liu J. (2014). A Multiple-SNP Approach for Genome-Wide Association Study of Milk Production Traits in Chinese Holstein Cattle. PLoS ONE.

[2] Ibeagha-Awemu E.M., Peters S.O., Akwanji K.A., Imumorin I.G., Zhao X. (2016). High Density Genome Wide Genotyping-by-Sequencing and Association Identifies Common and Low Frequency SNPs, and Novel Candidate Genes Influencing Cow Milk Traits. Sci. Rep..

[3] Jiang L., Liu J., Sun D., Ma P., Ding X., Yu Y., Zhang Q. (2010). Genome Wide Association Studies for Milk Production Traits in Chinese Holstein Population. PLoS ONE.

[4] Du A., Zhao F., Liu Y., Xu L., Chen K., Sun D., Han B. (2022). Genetic Polymorphisms of PKLR Gene and Their Associations with Milk Production Traits in Chinese Holstein Cows. Front. Genet..

[5] Liang Y., Gao Q., Zhang Q., Arbab A.A.I., Li M., Yang Z., Karrow N.A., Mao Y. (2020). Polymorphisms of the ACSL1 Gene Influence Milk Production Traits and Somatic Cell Score in Chinese Holstein Cows. Animals.

[6] Li M., Gao Q., Wang M., Liang Y., Sun Y., Chen Z., Zhang H., Karrow N.A., Yang Z., Mao Y. (2020). Polymorphisms in Fatty Acid Desaturase 2 Gene Are Associated with Milk Production Traits in Chinese Holstein Cows. Animals.

[7] Fu Y., Jia R., Xu L., Su D., Li Y., Liu L., Ma Z., Sun D., Han B. (2022). Fatty Acid Desaturase 2 Affects the Milk-production Traits in Chinese Holsteins. Anim. Genet..

[8] Han B., Liang W., Liu L., Li Y., Sun D. (2017). Determination of Genetic Effects of ATF3 and CDKN1A Genes on Milk Yield and Compositions in Chinese Holstein Population. BMC Genet..

[9] Shi L., Liu L., Lv X., Ma Z., Yang Y., Li Y., Zhao F., Sun D., Han B. (2019). Polymorphisms and Genetic Effects of PRLR, MOGAT1, MINPP1 and CHUK Genes on Milk Fatty Acid Traits in Chinese Holstein. BMC Genet..

[10] Li S., Xie Y., Zhang W., Gao J., Wang M., Zheng G., Yin X., Xia H., Tao X. (2015). Interferon Alpha-Inducible Protein 27 Promotes Epithelial–Mesenchymal Transition and Induces Ovarian Tumorigenicity and Stemness. J. Surg. Res..

[11] Chen Y., Jiao B., Yao M., Shi X., Zheng Z., Li S., Chen L. (2017). ISG12a Inhibits HCV Replication and Potentiates the Anti-HCV Activity of IFN-α through Activation of the Jak/STAT Signaling Pathway Independent of Autophagy and Apoptosis. Virus Res..

[12] Papac-Milicevic N., Breuss J.M., Zaujec J., Ryban L., Plyushch T., Wagner G.A., Fenzl S., Dremsek P., Cabaravdic M., Steiner M. (2012). The Interferon Stimulated Gene 12 Inactivates Vasculoprotective Functions of NR4A Nuclear Receptors. Circ. Res..

[13] Liu N., Long Y., Liu B., Yang D., Li C., Chen T., Wang X., Liu C., Zhu H. (2014). ISG12a Mediates Cell Response to Newcastle Disease Viral Infection. Virology.

[14] Rosebeck S., Leaman D.W. (2008). Mitochondrial Localization and Pro-Apoptotic Effects of the Interferon-Inducible Protein ISG12a. Apoptosis.

[15] Shen B., Zhang L., Lian C., Lu C., Zhang Y., Pan Q., Yang R., Zhao Z. (2016). Deep Sequencing and Screening of Differentially Expressed MicroRNAs Related to Milk Fat Metabolism in Bovine Primary Mammary Epithelial Cells. Int. J. Mol. Sci..

[16] Yu H., Zhao Y., Iqbal A., Xia L., Bai Z., Sun H., Fang X., Yang R., Zhao Z. (2021). Effects of Polymorphism of the *GPAM* Gene on Milk Quality Traits and Its Relation to Triglyceride Metabolism in Bovine Mammary Epithelial Cells of Dairy Cattle. Arch. Anim. Breed..

[17] Jelen P. (1995). Handbook of Milk Composition. Int. Dairy J..

[18] Wu Y., Sun Y., Zhang Z., Chen J., Dong G. (2020). Effects of Peptidoglycan, Lipoteichoic Acid and Lipopolysaccharide on Inflammation, Proliferation and Milk Fat Synthesis in Bovine Mammary Epithelial Cells. Toxins.

[19] Kukula M., Kolarič L., Šimko P. (2020). Decrease of Cholesterol Content in Milk by Sorption onto β-Cyclodextrin Crosslinked with Tartaric Acid; Considerations and Implications. Acta Chim. Slovaca.

[20] Kolarič L., Šimko P. (2022). Application of β-Cyclodextrin in the Production of Low-Cholesterol Milk and Dairy Products. Trends Food Sci. Technol..

